# Accelerating deployment of offshore wind energy alter wind climate and reduce future power generation potentials

**DOI:** 10.1038/s41598-021-91283-3

**Published:** 2021-06-03

**Authors:** Naveed Akhtar, Beate Geyer, Burkhardt Rockel, Philipp S. Sommer, Corinna Schrum

**Affiliations:** Institute of Coastal Systems-Analysis and Modeling, Helmholtz-Zentrum Hereon, Geesthacht, Germany

**Keywords:** Atmospheric dynamics, Wind energy

## Abstract

The European Union has set ambitious CO_2_ reduction targets, stimulating renewable energy production and accelerating deployment of offshore wind energy in northern European waters, mainly the North Sea. With increasing size and clustering, offshore wind farms (OWFs) wake effects, which alter wind conditions and decrease the power generation efficiency of wind farms downwind become more important. We use a high-resolution regional climate model with implemented wind farm parameterizations to explore offshore wind energy production limits in the North Sea. We simulate near future wind farm scenarios considering existing and planned OWFs in the North Sea and assess power generation losses and wind variations due to wind farm wake. The annual mean wind speed deficit within a wind farm can reach 2–2.5 ms^−1^ depending on the wind farm geometry. The mean deficit, which decreases with distance, can extend 35–40 km downwind during prevailing southwesterly winds. Wind speed deficits are highest during spring (mainly March–April) and lowest during November–December. The large-size of wind farms and their proximity affect not only the performance of its downwind turbines but also that of neighboring downwind farms, reducing the capacity factor by 20% or more, which increases energy production costs and economic losses. We conclude that wind energy can be a limited resource in the North Sea. The limits and potentials for optimization need to be considered in climate mitigation strategies and cross-national optimization of offshore energy production plans are inevitable.

## Introduction

**Figure 1:**
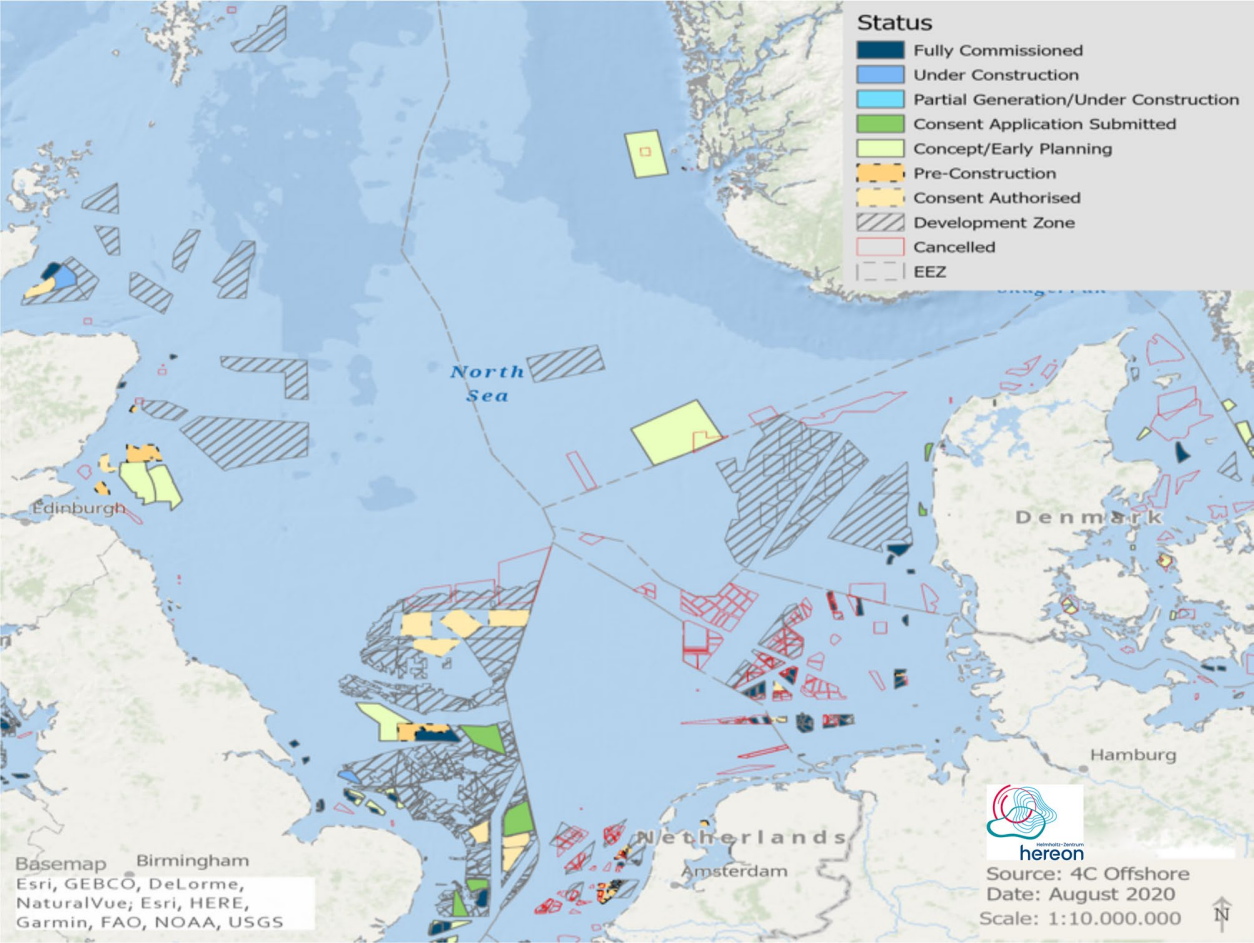
Distribution of OWFs in the North Sea (4c Offshore. https://www.4coffshore.com/windfarms/, 2019). Colors indicate the planning status of the OWFs by 2019. This map was created by Ulrike Kleeberg with ArcGIS Pro 10.7 (ESRI Inc. ArcGIS Pro 10.7, 2019).

The increasing demand for carbon–neutral energy production has fostered the rapidly increasing deployment of offshore wind farms (OWFs). The construction of OWFs is generally 1.5–2 times more expensive than onshore wind farms^1^. Additionally, their maintenance/repair, power network, and obtaining observational data for optimization are more challenging and costlier^2^. Although OWFs are more expensive in construction and maintenance than onshore wind farms, these costs are offset to some extent by the higher capacity factor (CF) of OWFs due to the strength of offshore wind resources^3^. About 10 km off the coast, sea surface winds are generally 25% higher than onshore winds. These high offshore wind resources can be utilized 2–3 times longer to generate electricity than onshore wind farms in the same period of time^4,5^. Europe’s total installed OWF capacity reached 22 GW in 2019; of that capacity, 77% is installed in the North Sea^6^. As part of the ambitious plans of the EU to reach climate neutrality a significant increase to 450 GW total offshore wind energy capacity is intended by 2050^7^. About 47% (212 GW) of these will be installed in the North Sea at an annual consenting rate of 8.8 GW per year during the 2020s^8^. This implies that the North Sea forms one of the worldwide hotspots of OWF development. Figure 1 shows the planning status of OWFs in the North Sea by 2019^9^. These massive developments are motivated by the strong and reliable wind resources in the North Sea at shallow water depths.

Wind farms are usually clustered around transmission lines to minimize deployment and operating costs. Hence, in addition to the quality of wind resources also the transmission lines determine whether a location is optimal for a wind farm. Despite the considerable availability of wind resources, evidence suggests that wake effects, which manifest as a downwind reduction in wind speed, can undermine the potential of cost-efficient wind energy production^10–12^. The efficiency limits that can arise from clustering and the overall regional saturation might limit the offshore wind energy production. These important questions at regional and longer times scales remain yet unassessed and need detailed scientific analysis for an efficient climate mitigation strategy. Additionally, in order to develop the OWFs efficiently and accurately, a comprehensive evaluation of the wind resources is required.

Wind turbines extract kinetic energy (KE) from the atmosphere and convert part of that energy into electric power. The remaining part of the energy is converted into turbulent kinetic energy (TKE); that generates wakes (downwind wind speed deficits)^13–17^. Airborne observations show that TKE is significantly increasing (factor of 10–20) above the wind farms^17^. These observations also show that wind farm wakes can extend up to 50–70 km under stable atmospheric conditions^18^. These wakes further impact the efficiency of downwind wind farms through changes in the temperature and turbulence in the boundary layer^19^. At a given wind speed, colder and denser air masses provide more energy than warmer and lighter air masses. Moreover, atmospheric turbulence additionally reduces the energy output and increases the load on wind farm structures and equipment^19^. Observational evidence shows that wakes can increase the temperature by 0.5 °C and humidity by 0.5 g per kilogram at hub height, even as far as 60 km downwind of wind farms^20^. Case studies related to wake dynamics have largely been limited to single wind turbines^21,22^ and/or individual wind farms^23–26^. Only a few studies have analyzed the wake effects caused by neighboring wind farms^11,25,27^. In a recent study^11^, the authors highlighted the economic losses suffered by onshore downwind wind farms due to the wake effects of upwind wind farms. Estimates of the wake effects on power production and environmental changes have been limited to short timescales (on the order of a few days or to a specific year^28^) and only one or two wind farms. The aforementioned studies emphasize the need to better understand the physical and economic interactions of large wind farms with complex clustered layouts (such as those planned in the North Sea) to ensure the efficient utilization of wind energy resources.

Building on process understanding of case studies, we assess for the first time the wake effect on the power production of both existing and planned large OWFs on a regional scale for the North Sea over a period of 10 years. It allows us to take into account the natural variability in wind climate, as inter-annual variability plays an important role in wind energy^29^. We perform two high-resolution numerical scenario simulations for a multi-year simulation period, one considering existing and currently planned OWFs in the North Sea and one for the undisturbed atmosphere. For the future scenario simulation, we apply a generic wind farm parameterization considering energy extraction and turbulence effects using a standard wind farm configuration, which we validate against earlier published high-resolution observations^30^ to ensure the realism of the scenario simulation. Mean wind changes will be analyzed and efficiency loss in offshore energy production will be estimated in terms of the Capacity Factor (CF) deficiency. Given the ongoing development of OWFs in the North Sea, our study highlights the urgent need to consider feedbacks between existing and planned OWFs to assess physical and economic impacts to optimize planning and to assess the limits and environmental impacts of industrial offshore energy production. To the best of our knowledge, this is the first study to estimate the wind speed deficits due to OWF production at a basin-wide scale covering a multi-year period and to investigate the effect of these deficits on the CF of wind farms. Furthermore, in this study, we evaluated the wind farm parameterization for real case simulations against the observations.

## Experimental design

All existing and planned OWFs by 2015^31^ in the North Sea area (see Fig. SI 1, the latest planning status is shown in Fig. 1) are considered for the scenario simulations. We focus on the Central and Southern North Sea where OWFs are planned close to each other. The scenario simulations are carried out for a multi-year period from 2008 to 2017, to account for a range of different weather conditions to assess the impact of large-scale OWF development on the production potential of wind farms. For the numerical simulations, we use the high-resolution Consortium for Small-Scale Modeling (COSMO)-CLimate Mode (CLM) regional climate model (RCM)^32^ both without and with a wind farm parameterization. An existing wind farm parameterization^15,16,33,34^ for a standard turbine size has been implemented into COSMO-CLM to include the effects of wind farms; these RCM simulations provide us with high-resolution spatiotemporal estimates of the wind speed over wind farm areas. A CF model^35^ has been used to assess the average energy production of wind farms based on wind speed. Several factors can influence the CF, such as the wake effect, turbine efficiency, and offshore distance^36^. For the inter-comparison of scenario simulations, we consider the impact of wakes on the CF, to illustrate the potential impact of feedbacks between wind farm deployment and regional atmospheric conditions. Hereafter, “CCLM_WF” and “CCLM” refer to the COSMO-CLM simulations with and without a wind farm parameterization, respectively.

## Verification of the simulated wind fields and OWFs wakes

### Comparison with the point observations of wind fields

**Figure 2:**
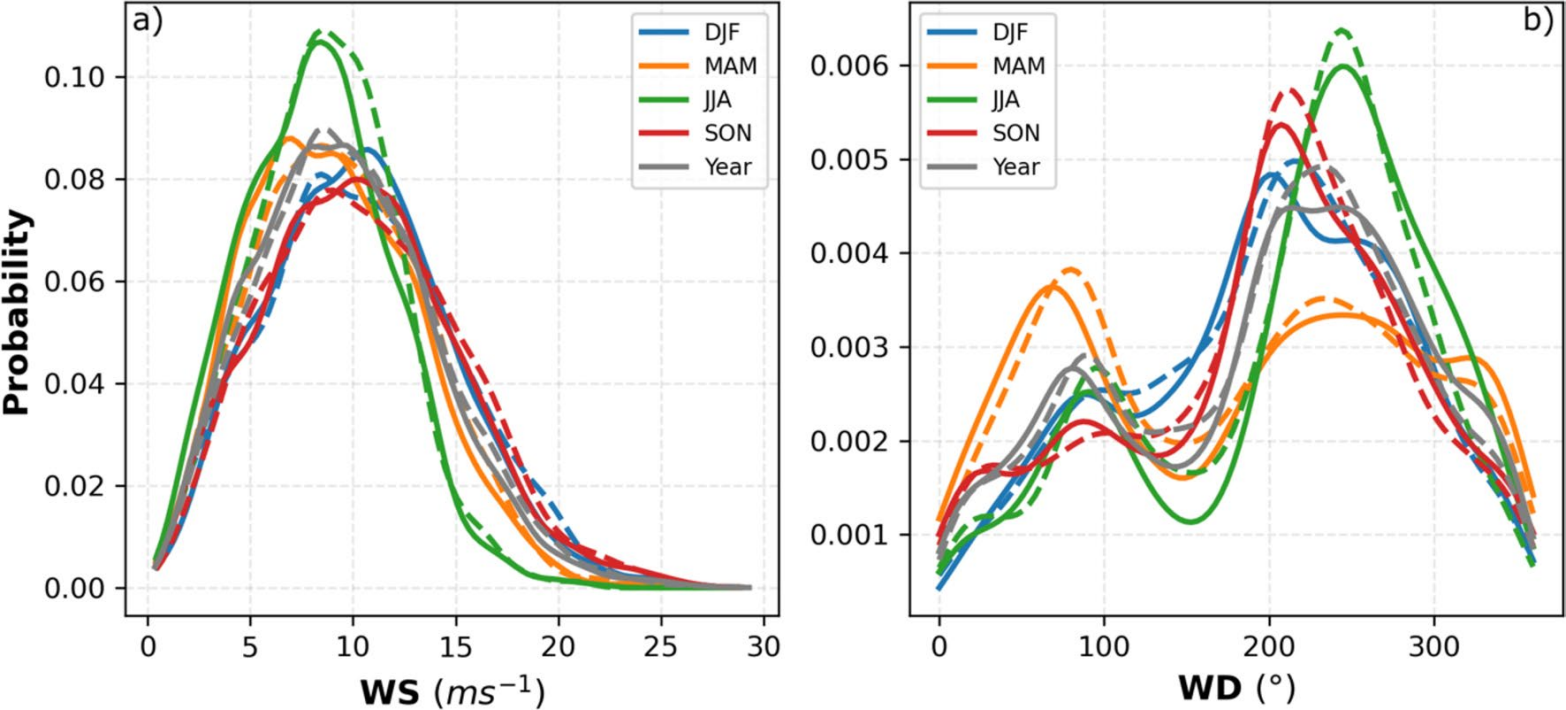
Annual and seasonal probability density functions calculated using the hourly (**a**) wind speed and (**b**) wind direction data at FINO1 (6.5875°E and 54.01472°N) at a height of 90 m in the period 2008–2009. Dashed lines result from measurements, while solid lines are from COSMO-CLM simulation. Gray lines indicate data for the entire period whereas colors indicate the different seasons as given in the legend.

To verify the realism of our scenario simulation, a detailed validation against published data^30,37^ was performed. The simulated wind characteristics over the North Sea can be directly evaluated using data from the research platforms^37^ FINO1 (6.5875°E, 54.01472°N) and FINO3 (7.158333°E, 55.195°N) starting in 2004 and 2009, respectively. The high quality of the mast-corrected measurement data allows for a detailed analysis of both the wind speed and the wind direction. Here we compared the FINO1 and FINO3 measurements with CCLM simulations for the period 2008–2009 and 2009–2014 respectively to avoid the effects of the OWF Alpha Ventus and DanTysk on the mast measurements^38^. The annual and seasonal probability density functions (PDFs) derived from hourly values of the wind speed and wind direction are in good agreement with the FINO1 data (Fig. 2). The annual and seasonal biases, root mean square error (RMSE), correlation coefficients, and Perkins’ score (PS)^39^ calculated between the CCLM simulation results and observations (FINO1 and FINO3) are presented in Tables 1 and 2. Compared with the FINO1 data, the CCLM winds show small, mostly negative biases of 0.27 ms^−1^ with simulated wind speeds that are lower than the observed wind speeds. During the spring and summer season model bias become stronger, along with higher RMSE values (Table 1). The autumn correlations of 0.87 are slightly higher than those in the other seasons. The PS of the yearly mean simulated wind speed is 0.95, with the highest values during winter (0.92) and the lowest values during summer (0.79). The simulated CCLM wind direction PDFs are also well represented; the prevailing southwesterly (200°–280°) wind directions are effectively captured (Fig. 2). On average, the CCLM-simulated wind directions show a positive bias of 3.07°, a small counterclockwise shift with an RMSE of 70.11° and a correlation coefficient of 0.71 (Table 1). Again, the simulated summer values show larger deviations from the observations with a bias of 8.08° and an RMSE of 72.18°; in addition, the correlation coefficient is lower than those in the other seasons. The simulated wind direction shows the highest PS during winter (0.88) and the lowest PS during spring (0.77) with a yearly value of 0.92. The simulated wind direction relative to FINO3 shows a negative bias of − 6.34°, an RMSE of 67.01°, a correlation coefficient of 0.75, and a PS of 0.93 (Table 2). Studies show that the existing wind farms in the North Sea are already affecting the wind field reaching FINO1 and FINO3^38^. A comparison of the wind speed and direction between CCLM_WF and FINO1 shows that the construction of planned wind farms will further affect their measurements in the future (Fig. SI 2). The annual and seasonal probability density functions (PDFs) derived from hourly values of the wind speed and wind direction are also in good agreement with the FINO3 data (Table 2 and Fig. SI 3).

**Table 1:** Yearly and seasonal mean wind speed and wind direction bias (CCLM – FINO1), root mean square error (RMSE), correlation (CORR), and Perkin’s score (PS) of CCLM compared with FINO1 in the period 2008–2009.

	Bias		RMSE		CORR		PS	
	WS (ms^−1^)	WD (°)	WS (ms^−1^)	WD (°)	WS	WD	WS	WD
Yearly	− 0.27	3.07	2.81	70.11	0.79	0.71	0.95	0.92
DFJ	− 0.08	1.44	3.41	64.61	0.73	0.71	0.92	0.88
MAM	− 0.34	1.21	2.54	76.53	0.82	0.72	0.85	0.77
JJA	0.40	8.08	2.73	72.18	0.72	0.67	0.79	0.80
SON	− 0.25	1.40	2.49	65.89	0.87	0.73	0.91	0.87

**Table 2:** Yearly and seasonal mean wind speed and wind direction bias (CCLM – FINO3), root mean square error (RMSE), correlation (CORR), and Perkin’s score (PS) of CCLM compared with FINO3 in the period 2009–2014.

	Bias		RMSE		CORR		PS	
	WS (ms^−1^)	WD (°)	WS (ms^−1^)	WD (°)	WS	WD	WS	WD
Yearly	− 0.39	− 6.34	2.59	67.01	0.85	0.75	0.95	0.93
DFJ	− 0.54	− 7.95	2.60	55.91	0.87	0.81	0.92	0.88
MAM	− 0.40	− 9.12	2.55	70.32	0.84	0.77	0.73	0.80
JJA	− 0.30	− 0.45	2.72	79.92	0.75	0.63	0.62	0.78
SON	− 0.37	− 7.99	2.50	58.11	0.85	0.79	0.81	0.89

### Wake effects in case studies: evaluation of CCLM

For the sake of completeness, CCLM_WF has been evaluated against airborne campaign data^18^ to illustrate the ability of CCLM_WF to simulate upwind flow and the spatial extent of wakes generated by wind farms. Here, we choose two different cases. In the first case, we evaluate the wake extent of the Amrumbank West wind farm; in the second case, we evaluated the wind speed deficit over the two Godewind farms. Only operational wind farms at the measurement times are considered in these simulations. Figure SI 4 shows the model domain and the wind farm locations.

#### Case 10 September 2016

A detailed comparison is performed for the wakes observed downwind of the Amrumbank West, Meerwind SüdOst, and Nordsee Ost wind farms with model simulations. The wake was measured during an aircraft campaign on 10 September 2016 between 0800 to 1100 UTC using five flight legs of 5 km, 15 km, 25 km, 35 km, and 45 km downwind of the Amrumbank West wind farm^18,30^. Stable atmospheric conditions and a wake extent of at least 45 km were measured. The installed turbines in Amrumbank West have a 90 m hub height and 120 m rotor diameter^12^. For this experiment, we employ only those wind farms which were existing at the time of measurements (see Fig. SI 4).

**Figure 3:**
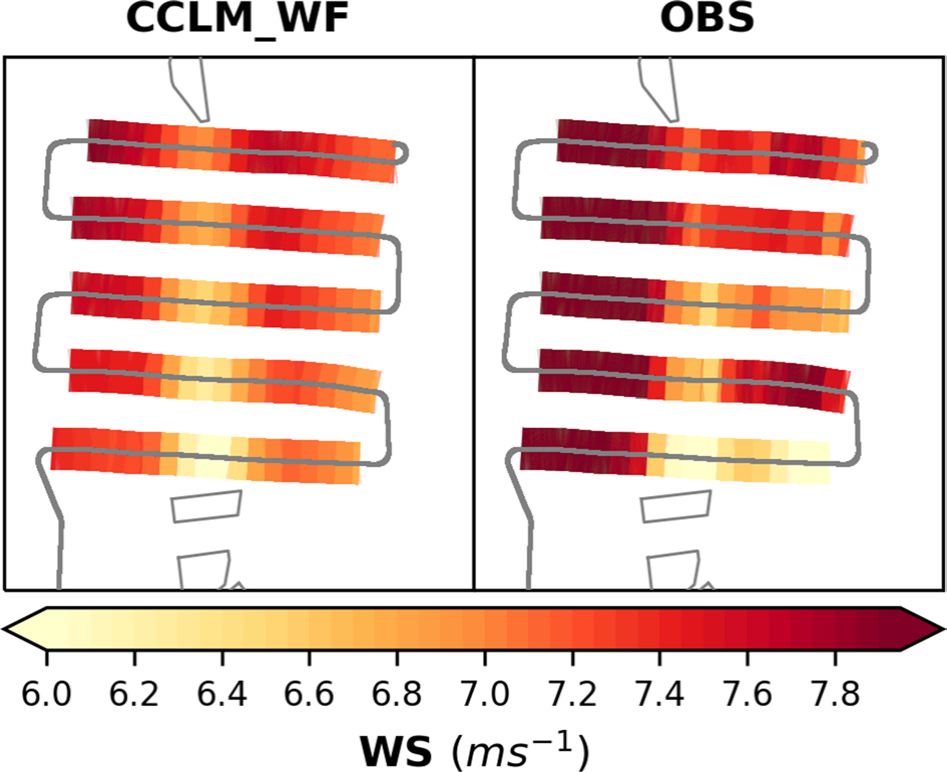
Wind speed at 90 m hub height (**a**) simulated in CCLM_WF and (**b**) observed by aircraft measurements. The aircraft track (gray lines) shown here ranged from 0820 to 0924 UTC on 10 September 2016. The model simulations show the wind speed at 0900 UTC.

The simulated spatial extent of the wake agrees well with the measurement. Figure 3 shows the wake extents simulated in CCLM_WF (interpolated on the aircraft track) and airborne observations (see Fig. SI 5a for a complete snapshot of the wind speed field simulated in CCLM_WF and its difference from the observation). Both the observations and the simulations show a wake extending more than 45 km downwind of the wind farm. The simulation shows that the wake reached down to the Butendiek wind farm, located 50 km downwind of the Amrumbank West wind farm. However, the simulated wind direction is slightly rotated counterclockwise. Similar to the width of the wind farms, the wake width is approximately 12 km at the beginning, which expands and weakens as the distance increases from the generating wind farm. The transect of the simulated and observed wind speeds through the first flight leg of 5 km downwind of the wind farm shows that the simulated-observed differences are smaller inside the wake than outside (Fig. SI 5b). In general, the model slightly underestimates the wind speed compared to the observations.

#### Case 14 October 2017

In the chosen case, we evaluate the wind speed at a height of 250 m over Godewind farms 1 and 2 with aircraft observations. The installed turbines in these wind farms have a 110 m hub height and 153 m diameter^18^. For this experiment, we employ the wind farm location data as in Fig. SI 4; however, we used the turbine dimensions as installed in Godewind farms.

**Figure 4:**
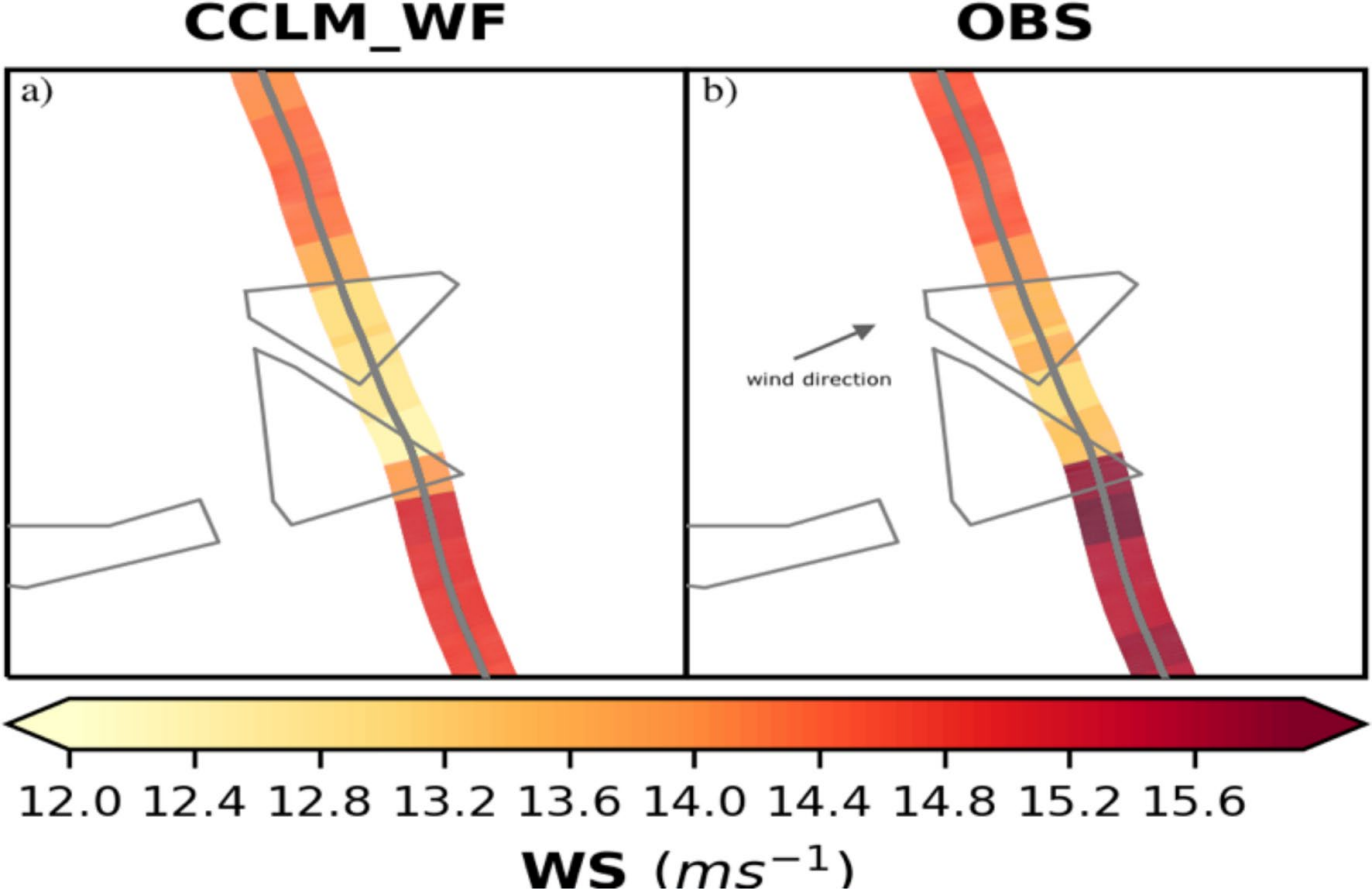
Wind speed at a height of 250 m (**a**) simulated in CCLM_WF and (**b**) observed by aircraft measurements. The aircraft track shown here ranged from 1445 to 1500 UTC on 14 October 2017. Arrow indicates the wind direction. The model simulations show the wind speed at 1500 UTC.

Figure 4 shows the wind speeds at 1500 UTC on 14 October 2017 over the Godewind farms simulated in CCLM_WF (interpolated on the aircraft track) and observed wind speeds (see Fig. SI 6a for a complete snapshot of the wind speed field simulated in CCLM_WF and its difference from the observation). Stable atmospheric conditions were observed at the times of the measurements^18^. An observed speed-up around the wind farms is well reproduced in the simulations. The simulated wind speeds agree better with the observations inside the wake than outside (Fig. SI 6b).

Due to the relatively coarse horizontal resolution of RCMs (1–2 km), the effects of individual wind turbines (with a rotor span of 120 or 153 m) cannot be fully resolved. Therefore, the simulated wake effects of the wind turbine can be underestimated, and thus, the wake effects of wind farms can be underestimated. In the present wind farm parameterization^16^, the power produced by the wind turbine depends on the wind speed in the grid cell at the model level interacting with the rotor. The wind turbine removes momentum from the rotor-interacting layers to produce the power that leads to wind speed deficits in downwind grid cells.

The evaluation results show that COSMO-CLM with a wind farm parameterization realistically reproduces the effects of wind farms. The spatiotemporal variability of the wake effects and their impact on the CF of the wind farms at 90 m hub height are analyzed for the period 2008–2017 in the following sections.

## Wake effect on wind speed and turbulent kinetic energy

**Figure 5:**
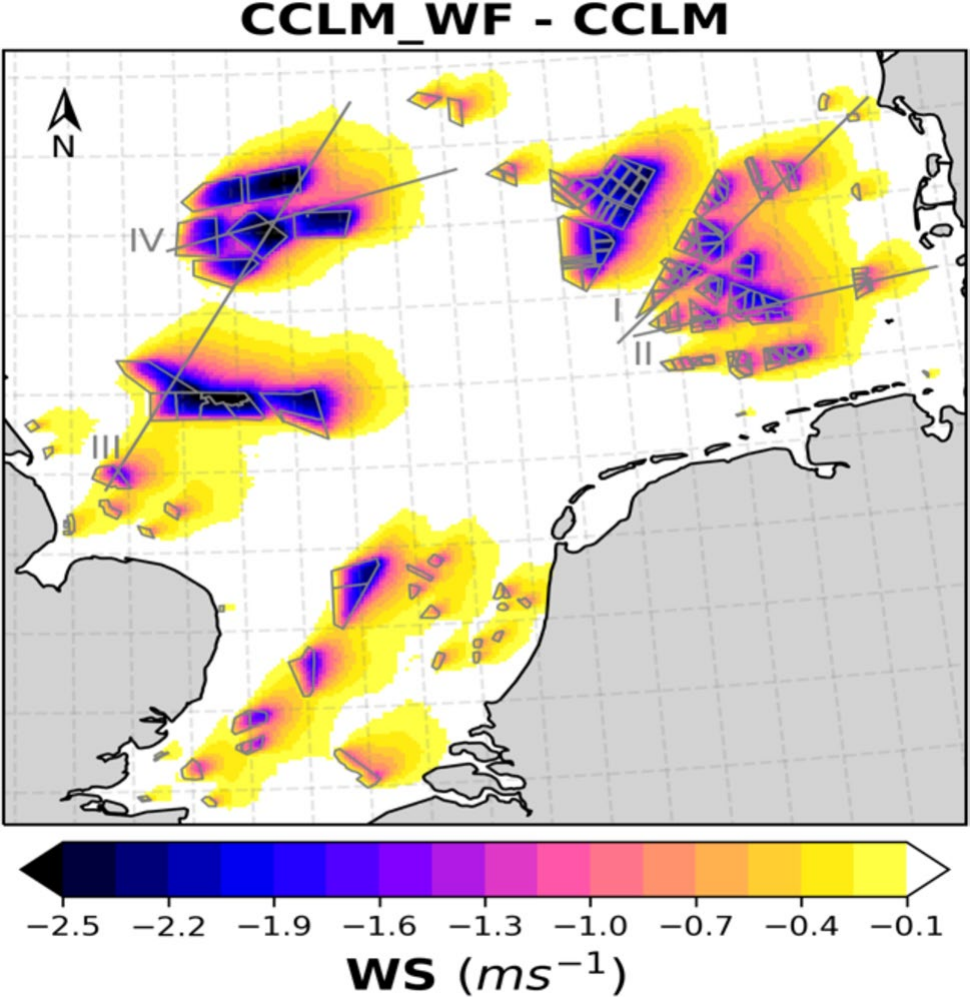
Annual mean wind speed deficits (CCLM_WF – CCLM) outside and inside the wind farms for the prevailing wind directions of 200°–280° at hub height (90 m) in the period 2008–2017. Numbered gray lines indicate the transects used for calculations of Fig. 8 and Fig. SI 6 and SI 7. This figure was created with Matplotlib (Hunter, J. D., Matplotlib: a 2D graphics environment. Computing in Science and Engineering* 9*, 2007) and Cartopy (Met office, Cartopy: a cartographic python library with a matplotlib interface. Exeter, Devon, https://scitools.org.uk/cartopy, 2015).

Our simulations show that the development of massive clustered OWFs significantly impacts the wind climate and efficiency of renewable energy production on a regional scale. The reduction in the annual mean wind speed reaches up to 2–2.5 ms^−1^ during prevailing southwesterly (200°–280°) winds, and that in the seasonal mean reaches more than 3 ms^−1^ (see Fig. 5 and Figs. SI 2 and SI 8).

**Figure 6:**
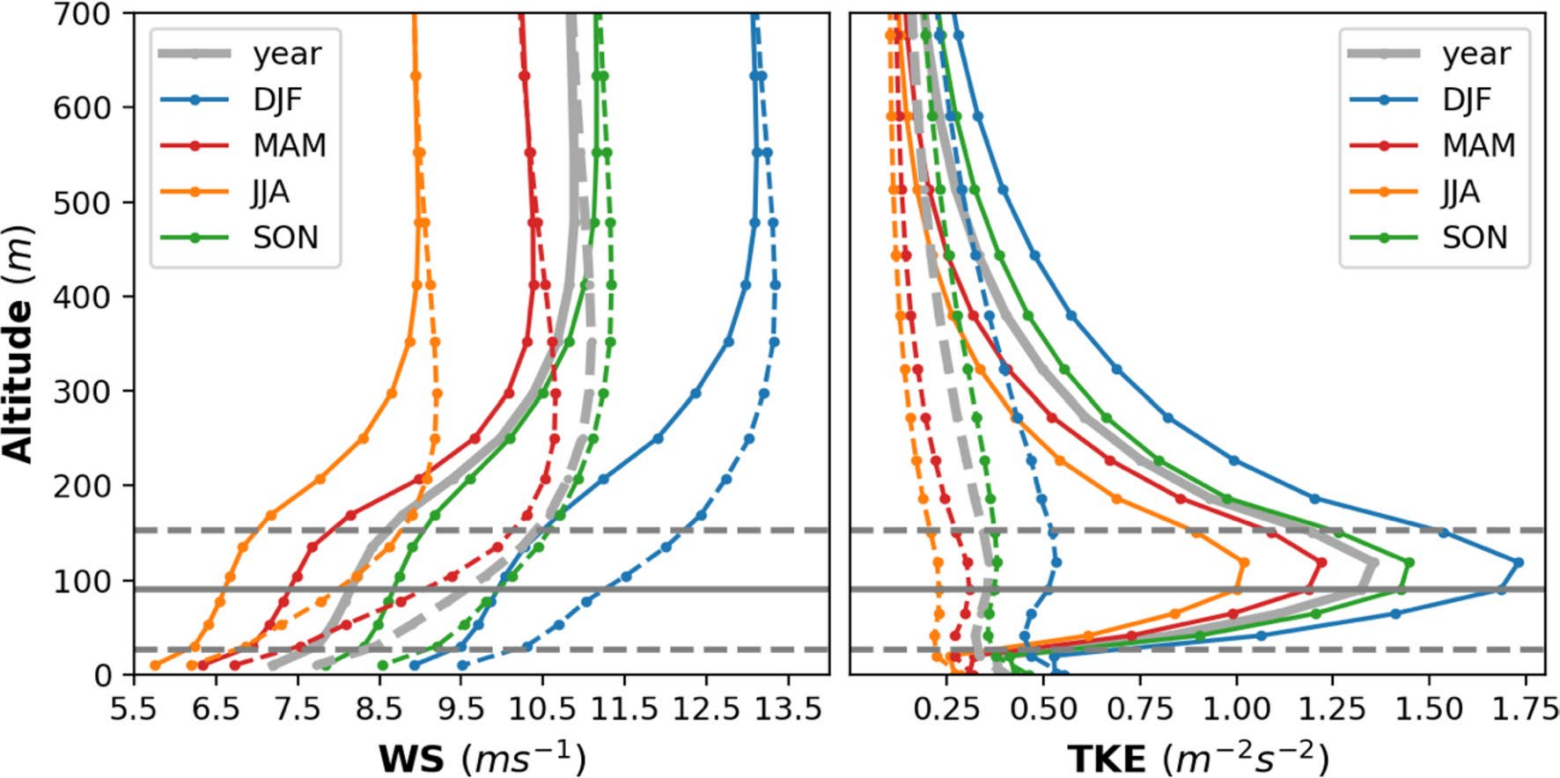
Annual and seasonal mean vertical profiles of the wind speed (left) and turbulent kinetic energy (right) simulated by CCLM (broken dotted lines) and CCLM_WF (solid dotted line) over the wind farm areas in the period 2008–2017. Solid circles indicate the model levels. The horizontal solid gray line indicates the hub height (90 m) of the turbine whereas dotted gray lines indicate lower (27 m) and upper (153 m) tip of the rotor.

The wind speed in the North Sea exhibits strong spatial and temporal variability. At 90 m hub height, the wind speed varies seasonally, with a minimum of approximately 7–8.5 ms^−1^ in summer and a maximum of 10–11.5 ms^−1^ in winter (Fig. SI 7). The presence of a wind farm impacts the boundary layer flow over the wind farm and its vicinity by extracting KE from the mean flow and generating TKE. The highest wind speed deficit in the annual mean is about − 18%, and the increase in TKE is nearly a factor of 4 over the wind farm itself (Fig. 6). These changes in wind speed and TKE extend vertically to a height of approximately 500 m (about 350 m above the turbine height). A deficit/raise of about 1 ms^−1^/0.6 m^−2^ s^−2^ in wind speed/TKE extends to a height of approximately 200 m. The maximum change in wind speed and TKE found in the atmospheric levels between the hub (90 m) and tip height (153 m) of the wind turbines. The change in the wind speed and TKE above the turbine height is consistent with the previous studies^16,40,41^. The wind speed deficits are higher during spring (− 22%) and summer (− 20.8%) than during the other seasons (see also Fig. SI 8), the reason for which is explained later in this section. The increase in the TKE is found higher during winter (factor of 3.2) and autumn (factor of 3.8). The addition TKE source in the wind farm parameterization improves the representation of mixing and wind speed deficit during stable conditions^17^. The change in wind speed and TKE increases the boundary layer height^16^.

Wakes, i.e., downwind reductions in wind speed, exhibit significant spatial variability inside and outside wind farms (Fig. 5). The wind speed deficit inside a wind farm increases with increasing distance from the upstream edge, reaching a maximum of 2–2.5 ms^−1^. In an idealized numerical study, a maximum reduction of approximately 16% in the wind speed and increase in TKE by nearly a factor of 7 was estimated at hub height over a 10 × 10 km wind farm^16^. Here we used a realistic climate set up to study a scenario with clustered and large-scale wind farms and found larger mean wind speed deficits of approximately 18–20% of the annual mean wind. In our case, the increase in the mean TKE within the wind farm is almost a factor of 3 less than that reported (factor of 7) in the idealized study^16^. This could be due to the reason that mean values of TKE over a longer period 2008–2017 are shown here.

The wind farm induced boundary layer mixing, air friction, turbulence and weaken stratification effects within and above the rotor area that reach about 600 m. The maximum differences are found in the layers between the hub and tip height of the turbine. The reduction in the wind speed extends highest during spring when the atmospheric conditions are generally stable. The increase in TKE leads to the mixing of more momentum from aloft^15,24^. This mechanism is more pronounced during winter and autumn when atmospheric conditions are generally unstable in the North Sea. The strength of the TKE depends on the difference between the power coefficient and thrust coefficients which varies with the wind speed.

The wakes forming downwind extend over large distances and influence the wind climate at surrounding wind farms. The wake extends varies, it depends on wind speed and atmospheric stratification and might extend up to 70 km downwind^11,18,20^. On average wakes extend ca 40–45 km downwind (Fig. SI 8).

## Implications for the CF

**Figure 7:**
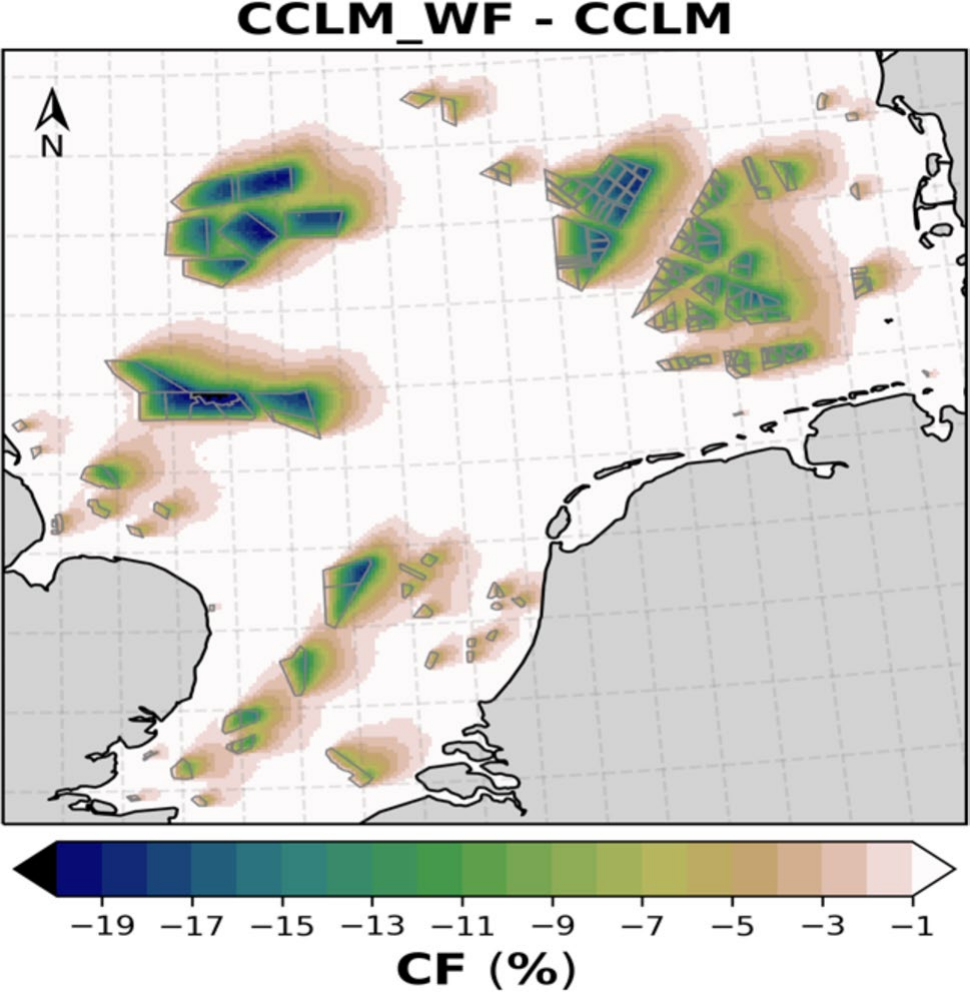
Annual mean losses in the capacity factor CF (CCLM_WF – CCLM) out- and inside of the wind farms (gray lines) for the prevailing wind directions of 200°–280° at hub height (90 m) in the period 2008–2017. This figure was created with Matplotlib (Hunter, J. D., Matplotlib: a 2D graphics environment. Computing in Science and Engineering* 9*, 2007) and Cartopy (Met office, Cartopy: a cartographic python library with a matplotlib interface. Exeter, Devon, https://scitools.org.uk/cartopy, 2015).

The downwind speed reduction results in a significant decrease in the efficiency of energy production here illustrated in terms of the CF. The wake induced decrease in CF up to 22% in the annual mean and up to 26% for the seasonal mean with the highest values at the downwind edge within the wind farms during southwesterly wind directions (see Fig. 7 and Figs. SI 4 and SI 5). Outside of the wind farms, these values decrease as the distance from the wind farms is increasing. A decrease of about 1% has been noted at a distance of 35–40 km in annual means during southwesterly wind directions. The highest drops are observed for the large wind farms in the German Bight and the UK’s Dogger Bank for southwesterly wind directions (Fig. 7).

Without the wind farms, the annual mean CF for all wind directions varies spatially in the North Sea from 50 to 62%, with higher values during winter (65–70%) and lower values in summer (37–50%, Fig. SI 9). These values are strongly reduced in the areas where the large-size wind farms are clustered. The mean wind speed deficits and CF losses for all wind directions show that the wake effect extends more towards the northeast than in the other wind directions, indicating the dominance of southwesterly winds (Fig. SI 8 and Fig. SI 10).

**Figure 8:**
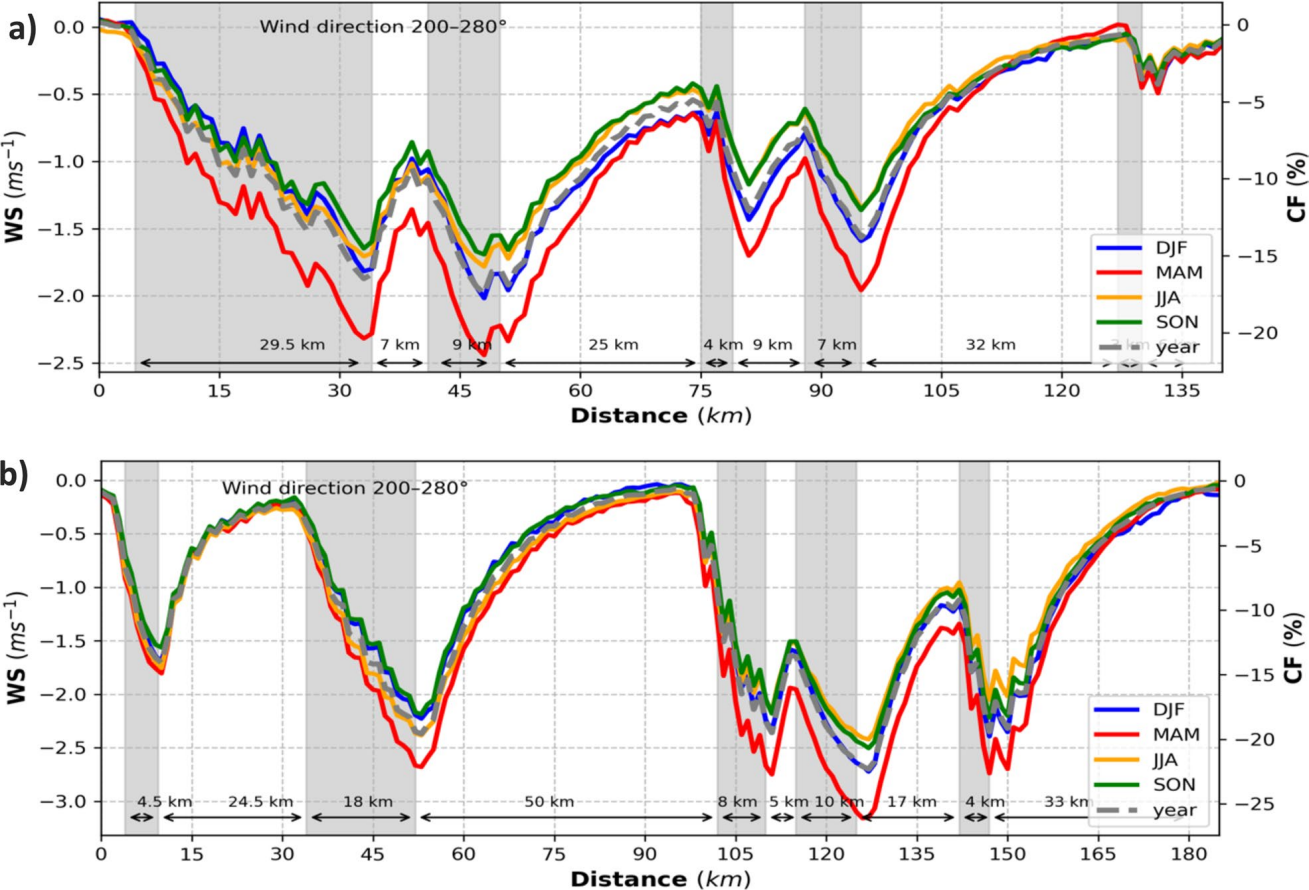
(**a**) Transects of the seasonal (colored, see legend) and yearly mean (dashed gray) wind speed deficits (left axis; CCLM_WF – CCLM) and capacity factor losses (right axis; CCLM_WF – CCLM) for the prevailing wind directions of 200°–280° in the period 2008–2017 at hub height (90 m) taken at transect I (German Bight, Fig. 5) latitude 54.2 latitude 54.2°N–55.6°N and longitude 5.45°E–8.0°E. Gray sectors indicate the wind farm positions. Arrows and attached numbers give the distances between the edges of the wind farms. (**b**) As of (**a**) but for transect III (Dogger Bank, Fig. 5) latitude 54.4°N–55.8°N and longitude 0.8°E–3.15°E.

A more specific analysis of the implications of large wind farm clusters and extremely large farms for the efficiency of neighboring farms and clusters in the area of the German Bight and the Dogger Bank (Fig. SI 1) highlights substantial CF losses. Figure 8 shows the annual and seasonal mean wind speed deficits and CF losses through the wind farms on two of the transects (gray lines I and III) shown in Fig. 5 in the case of prevailing winds in the German Bight and the UK’s Dogger Bank. The wind farms in both of these areas are large and are located spatially close to each other. These transects show the strong horizontal influences of the wind farms together with the reductions in the wind speed and CF. Mean CF and wind speed show characteristic patterns along transects crossing several wind farms (Fig. 8). The wind speed deficit, being higher towards the downwind wind farm edge, leads to an annual reduction of up to 25% in the CF of downwind wind turbines inside wind farms; outside these wind farms, the CF losses reach up to 20% depending on the size of the farm and distance away from it. For example, as shown in Fig. 8a, wind farm 2, which is 7 km from wind farm 1, suffers a mean wind speed deficit of 1–1.5 ms^−1^. This reduces the CF of upwind turbines by 10–15% and that of downwind turbines by 15–20% in wind farm 2. Then, the wakes generated by wind farm 2 extend up to wind farm 3 (25 km away) with a deficit of 0.5–0.8 ms^−1^ and CF losses of 5–8%. The wake effect of wind farm 4 reaches up to 30 km. The wind speed between wind farms 1 and 2 recovers approximately 45% in 5 km. However, the recovery of the wind speed in the following wind farms is slow due to the accumulated effects. Similarly, as shown in Fig. 8b, the wake effect reaches approximately 33 km between wind farms 2 and 3 and approximately 28 km beyond wind farm 5. The wake generated by the wind farm 4 reduces the CF of wind farm 5 (17 km away) up to 12%. Due to the short distance between wind farms 3 and 4 (about 5 km), wind farm 4 receives about 1.5–2 ms^−1^ less wind speed which is equivalent to CF losses of 12–16%, during prevailing southwesterly winds. The transects of lines II and IV are shown in Fig. SI 11. The most productive wind turbines/farms are those located on the grid-cells at upwind edge/farms of the wind farms where the wind flow is uninterrupted^25^.

The wake effect can substantially influence the economic potential of wind power generation within a cluster, in large farms, and in neighboring farms located at a distance within the wake. Annual mean wind speed deficits of 1–1.5 ms^−1^ and CF deficits of wind farms in the vicinity of large downwind clusters are frequent, within clusters, the reduction is even stronger and amounts up to a seasonal mean wind speed reduction of more than 3 ms^−1^ or a seasonal CF reduction of up to 25% (Fig. 8). Average wakes extend up to 40 km for the largest wind farms and clusters.

The highest wind speed deficits occur during the spring season which leads to the highest CF losses in these seasons. On a monthly timescale, the highest wind speed deficits are simulated in March and April, whereas the lowest deficits are simulated in November and December (see Fig. SI 12). The seasonal variations in wind speed deficits are related to the relatively stronger winds (see Fig. SI 12) and weaker stratification^42^ during the autumn and winter seasons compared to the spring seasons. During spring, the atmospheric conditions are more stable than the other seasons which leads to longer wakes^18,42–44^. It implies that the most productive season is winter when the wind speed is higher and the stratification not stable.

## Discussion and conclusions

The results show that the wind fields simulated by the regional climate model COSMO-CLM are in good agreement with the mast measurement stations FINO1 and FINO3 in the North Sea. It also indicates that the deployment of large wind farms near the mast measurement stations will affect their measurements. The COSMO-CLM model with the wind farm parametrization^15^ simulated the wake generated by the wind farms reasonably well. Despite the differences in the upwind wind speed, the length and width of the wake were simulated quite well.

Our results show that clusters of large wind farms, such as the farms planned for the near future in the UK’s Dogger Bank and the German Bight, have the potential to substantially modify the atmospheric dynamics and lead to local mean wind speed reductions extending as far as more than 40 km downwind from the farm. Depending on the size of the wind farm, generally, the annual mean wind speed deficit can reach 2–2.5 ms^−1^ which is equivalent to the power loss of 1–2 MW^45^. These results are consistent with the previous studies^15,46,47^. These authors studied the consequences of wind farms in case studies and short-term simulations. Our results show that the previously identified effects accumulate and influence the mean wind pattern. We identified a trade-off in the clustering of offshore wind farms. Clustering supports reduced energy production costs due to reduced infrastructure investments, but these advantages can be offset by wakes effects and the consequent reduction of CF. Our results emphasize that wind energy in the North Sea can be considered a limited resource. With the current plans to install offshore wind energy farms in the North Sea locally resource exploitation limits are reached. Better planning and optimization of locations are required that consider the development of wind wakes under realistic multi-year atmospheric conditions.

It is important to note that for our idealized study we used an average size (90 m hub height and 126 m rotor diameter) of turbines for existing wind farms. The rapidly increasing size and power generation of wind turbines^48^ can intensify the wake effects vertically and horizontally. Moreover, wind farm installations in the North Sea are further accelerating and the here identified limits of power generation will become more important.

Southwesterly winds are predominant in the North Sea^49^ (Fig. 2 and Fig. SI 3), and wake effects and their implications for power generation are therefore of particular importance for efficient energy production and production costs. During prevailing southwesterly winds, the power production of a downwind wind farm on the northeastern side is generally undermined by the wind farms located upwind.

Under stably stratified atmospheric conditions, weak vertical momentum mixing strengthens the wake effect^11,15,18,20^, and observational evidence shows that the wake can extend up to 50–70 km under such atmospheric conditions^30^. Such individual cases are also well reproduced in the model simulations. These findings suggest that CF losses can be greater than the mean values shown herein and last longer under stable atmospheric conditions. Additionally, this study shows the annual and seasonal mean values calculated using hourly values during the period 2008–2017 to illustrate the mean wake effect on the CF using multi-year weather conditions under all atmospheric conditions. This shows that the wind speed and CF deficits are highest during spring (mainly March–April) and lowest during November–December. The proximity of large wind farms affects the production of downwind wind turbines and wind farms, reducing the CF by more than 20–25%.

Already now, offshore renewable energy production in the North Sea shows substantial impacts on the atmospheric conditions therein, and these effects will continue to increase in the future. The evidence indicates that OWFs can impact marine animals and can raise environmental and climate concerns^2,50,51^. Since wind is one of the main factors modulating ecosystem productivity and ecosystem structure, OWFs have the potential to develop into dominant ecosystem drivers and need to be considered for ecosystem management and fisheries assessment. Therefore, an optimization strategy based on both national and international considerations is required to minimize economic losses and to assess the limits and environmental impacts of industrial offshore energy production. Furthermore, atmospheric wakes can induce ocean responses by modifying the sea surface roughness, atmospheric stability, and heat fluxes, and hence have the potential to influence local climate that requires further investigation^32,52,53^.

## Methods

### Numerical model setup

In this study, we employ the regional climate model COSMO-CLM^32^ with a wind farm parameterization^15,33,34^ to consider the wind farm impacts on local atmospheric dynamics and the spatial–temporal pattern of wind speed deficits for a near-future wind farm scenario in the North Sea (see Fig. SI 1). COSMO-CLM uses a horizontal atmospheric grid mesh size of 0.02° (~ 2 km; 396 × 436 grid cells) and 62 vertical levels. In our configuration, COSMO-CLM uses a time step of 12 s with a third-order Runge–Kutta numerical integration scheme. The physics options include a cloud microphysics scheme, a delta-two-stream scheme for shortwave and longwave radiation, and a one-dimensional prognostic TKE advection scheme for the vertical turbulent diffusion parameterization^54^. The roughness length over the sea is computed on the basis of the Charnock formula^54^. The initial and lateral boundary conditions for the wind, sea surface temperature and other meteorological variables are taken from a CoastDat3 simulation^29^, which provides hourly data at a horizontal resolution of 0.11° (~ 11 km). The CoastDat3 atmospheric simulation was driven by European Centre for Medium-Range Weather Forecast (ECMWF) ERA-Interim reanalysis data in 6 hourly intervals at a horizontal resolution of 0.703°^55^.

To include wind farm effects, a wind farm parameterization for mesoscale numerical weather prediction models is implemented into COSMO-CLM^56^. This parameterization represents wind turbines as a momentum sink for the mean flow that converts KE into electric energy and TKE. The parameterization uses the velocity in each grid to estimate the average effect of the wind turbines within that grid. In our configuration, we use five vertical levels within the rotor area. The wind turbine extracts KE from the mean flow of each layer intersecting the rotor area. The amount of extracted KE depends on the wind speed, thrust, power coefficients, air density, and the density of the wind turbines in the considered grid^45^ (see Fig. SI 13). A fraction of the extracted KE is converted into electric power by the turbine, whereas the remaining part of KE is converted into TKE. Here, we use the thrust and power coefficients as a function of wind speed derived from the theoretical National Renewable Energy Laboratory (NREL) 5 MW reference wind turbine for offshore system development^45^. These coefficients are close to those of real wind turbines, as the NREL 5 MW turbine data were derived from the REPower 5 MW offshore wind turbine. The wind turbine is hallmarked by a cut-in wind speed of 3 ms^−1^, a rated power speed of 12 ms^−1^, and a cut-out speed of 25 ms^−1^. In this study, we used the 90 m hub height and a 126 m rotor diameter with a rated power of 5.3 MW. The chosen turbine size falls within the range of existing wind farms by 2017 (Table SI 3). For a more detailed description of the wind farm parameterization and its implementation, we refer the readers to the previous studies^15,33,34^.

### Capacity factor (CF)

Because of the high variability of wind, low, medium, and high wind speeds alternate frequently, and wind turbines cannot operate continuously at the rated power. Therefore, the CF is commonly used to calculate the average energy production of a wind turbine. In turn, the CF is used for the economic assessment of a project, optimum turbine site matching, and the ranking of potential sites^35^. Several generic models are available in the literature to represent the ascending segment of the power curve between the cut-in and rated speeds (Fig. SI 13) independent of the power coefficients, which are unique to every turbine and difficult to generalize. These generic models use the cut-in, rated, and cut-out speeds to estimate the ascending segment of the power curve without information on the turbine output. We use a polynomial generic model^35^ to estimate the CF using a Weibull probability density function based on hourly wind speed values and three speeds, namely, the cut-in (3 ms^−1^), rated (12 ms^−1^), and cut-out (25 ms^−1^), of the performance curve shown in Fig. SI 13.

## Supplementary Information

Supplementary Figures.

## Data Availability

The model COSMO-CLM_WF and COSMO-CLM datasets supporting the results can be downloaded via CERA-DKRZ^57,58^ and the COSMO-CLM namelists are available from the authors upon request. The COSMO-CLM simulations employ the community-wide publicly available (http://www.clm-community.eu) COSMO-CLM code. In situ airborne observational data were accessed via PANGAEA^30^ and the FINO data were obtained via https://www.fino-offshore.de/en/ and http://fino.bsh.de.

## References

[1] Zheng CW, Li CY, Pan J, Liu MY, Xia LL (2016). An overview of global ocean wind energy resource evaluations. Renew. Sustain. Energy Rev..

[2] Leung DYC, Yang Y (2012). Wind energy development and its environmental impact: A review. Renew. Sustain. Energy Rev..

[3] Junfeng, L., Pengfei, S. & Hu, G. China Wind Power Outlook 2010 (2010).

[4] Tambke J, Lange M, Focken U, Wolff JO, Bye JAT (2005). Forecasting offshore wind speeds above the North Sea. Wind Energy.

[5] Wang J, Qin S, Jin S, Wu J (2015). Estimation methods review and analysis of offshore extreme wind speeds and wind energy resources. Renew. Sustain. Energy Rev..

[6] WindEurope. Offshore wind in Europe: Key trends and statistics 2019 (2019).

[7] The European Green Deal. Communication from the commission to the European parliament, the European council, the council, the European economic and social committee and the committee of the regions (2019).

[8] WindEurope. Our Energy Our Future: How offshore wind will help Europe go carbon-neutral (2019).

[9] 4c Offshore. https://www.4coffshore.com/windfarms/. (2019).

[10] Hasager CB (2017). Wind farm wake: The 2016 Horns Rev photo case. Energies.

[11] Lundquist JK, DuVivier KK, Kaffine D, Tomaszewski JM (2019). Costs and consequences of wind turbine wake effects arising from uncoordinated wind energy development. Nat. Energy.

[12] Siedersleben SK (2018). Evaluation of a wind farm parametrization for mesoscale atmospheric flow models with aircraft measurements. Meteorol. Z..

[13] Rhodes ME, Lundquist JK (2013). The effect of wind-turbine wakes on summertime US midwest atmospheric wind profiles as observed with ground-based Doppler Lidar. Bound.-Layer Meteorol..

[14] Djath B, Schulz-Stellenfleth J, Cañadillas B (2018). Impact of atmospheric stability on X-band and C-band synthetic aperture radar imagery of offshore windpark wakes. J. Renew. Sustain. Energy.

[15] Fitch AC, Olson JB, Lundquist JK (2013). Parameterization of wind farms in climate models. J. Clim..

[16] Fitch AC (2012). Local and mesoscale impacts of wind farms as parameterized in a mesoscale NWP model. Mon. Weather Rev..

[17] Siedersleben SK (2020). Turbulent kinetic energy over large offshore wind farms observed and simulated by the mesoscale model WRF (3.8.1). Geosci. Model Dev..

[18] Platis A (2018). First in situ evidence of wakes in the far field behind offshore wind farms. Sci. Rep..

[19] Irena. Renewable energy technologies: Cost analysis series. *Green Energy Technol*. **1** (2012).

[20] Siedersleben SK (2018). Micrometeorological impacts of offshore wind farms as seen in observations and simulations. Environ. Res. Lett..

[21] Smalikho IN (2013). Lidar investigation of atmosphere effect on a wind turbine wake. J. Atmos. Ocean. Technol..

[22] Churchfield MJ, Lee S, Michalakes J, Moriarty PJ (2012). A numerical study of the effects of atmospheric and wake turbulence on wind turbine dynamics. J. Turbul..

[23] Aitken ML, Kosović B, Mirocha JD, Lundquist JK (2014). Large eddy simulation of wind turbine wake dynamics in the stable boundary layer using the Weather Research and Forecasting Model. J. Renew. Sustain. Energy.

[24] Calaf M, Meneveau C, Meyers J (2010). Large eddy simulation study of fully developed wind-turbine array boundary layers. Phys. Fluids.

[25] Nygaard NG (2014). Wakes in very large wind farms and the effect of neighbouring wind farms. J. Phys. Conf. Ser..

[26] Nygaard NG, Christian Newcombe A (2018). Wake behind an offshore wind farm observed with dual-Doppler radars. J. Phys. Conf. Ser..

[27] Nygaard NG, Hansen SD (2016). Wake effects between two neighbouring wind farms. J. Phys. Conf. Ser..

[28] Badger, J. *et al*. Making the most of offshore wind: Re-evaluating the potential of offshore wind in the German North Sea. Study commissioned by Agora Energiewende and Agora Verkehrswende, 1–84 (2020).

[29] Geyer B, Weisse R, Bisling P, Winterfeldt J (2015). Climatology of North Sea wind energy derived from a model hindcast for 1958–2012. J. Wind Eng. Ind. Aerodyn..

[30] Bärfuss, K. *et al.* In-situ airborne measurements of atmospheric and sea surface parameters related to offshore wind parks in the German Bight. PANGAEA 10.1594/PANGAEA.902845 (2019).

[31] EWEA. The European offshore wind industry key 2015 trends and statistics. *… Documents/Publications/Reports/Statistics/ …* 31 (2015). 10.1109/CCA.1997.627749.

[32] Rockel B, Will A, Hense A (2008). The regional climate model COSMO-CLM (CCLM). Meteorol. Z..

[33] Chatterjee F, Allaerts D, Blahak U, Meyers J, van Lipzig NPM (2016). Evaluation of a wind-farm parametrization in a regional climate model using large eddy simulations. Q. J. R. Meteorol. Soc..

[34] Blahak, U., Goretzki, B. & Meis, J. A simple parameterization of drag forces induced by large wind farms for numerical weather prediction models. *European Wind Energy Conference and Exhibition 2010, EWEC 2010* **6**, 4577–4585 (2010).

[35] Albadi MH, El-Saadany EF (2010). Optimum turbine-site matching. Energy.

[36] Dean N (2020). Performance factors. Nat. Energy.

[37] Leiding, T. *et al.* Standardisierung und vergleichende Analyse der meteorologischen FINO-Messdaten (FINO123) (2016).

[38] Westerhellweg A, Cañadillas B, Kinder F, Neumann T (2014). Wake measurements at alpha ventus—Dependency on stability and turbulence intensity. J. Phys. Conf. Ser..

[39] Perkins SE, Pitman AJ, Holbrook NJ, McAneney J (2007). Evaluation of the AR4 climate models’ simulated daily maximum temperature, minimum temperature, and precipitation over Australia using probability density functions. J. Clim..

[40] Lu H, Porté-Agel F (2011). Large-eddy simulation of a very large wind farm in a stable atmospheric boundary layer. Phys. Fluids.

[41] Chamorro LP, Porté-Agel F (2009). A wind-tunnel investigation of wind-turbine wakes: Boundary-Layer turbulence effects. Bound. Layer Meteorol..

[42] Djath B, Schulz-Stellenfleth J (2019). Wind speed deficits downstream offshore wind parks—A new automised estimation technique based on satellite synthetic aperture radar data. Meteorol. Z..

[43] Emeis S (2010). A simple analytical wind park model considering atmospheric stability. Wind Energy.

[44] Christiansen MB, Hasager CB (2005). Wake effects of large offshore wind farms identified from satellite SAR. Remote Sens. Environ..

[45] Jonkman J, Butterfield S, Musial W, Scott G (2009). Definition of a 5-MW reference wind turbine for offshore system development. United States.

[46] Abkar M, Porté-Agel F (2015). Influence of atmospheric stability on wind-turbine wakes: A large-eddy simulation study. Phys. Fluids.

[47] Allaerts, D. Large-eddy simulation of wind farms in conventionally neutral and stable atmospheric boundary layers (2016).

[48] IEA (2019). Offshore Wind Outlook 2019. World Energy Outlook.

[49] Siegismund F, Schrum C (2001). Decadal changes in the wind forcing over the North Sea. Clim. Res..

[50] Saidur R, Rahim NA, Islam MR, Solangi KH (2011). Environmental impact of wind energy. Renew. Sustain. Energy Rev..

[51] Tabassum A, Premalatha M, Abbasi T, Abbasi SA (2014). Wind energy: Increasing deployment, rising environmental concerns. Renew. Sustain. Energy Rev..

[52] Boettcher M, Hoffmann P, Lenhart HJ, Heinke Schlunzen K, Schoetter R (2015). Influence of large offshore wind farms on North German climate. Meteorol. Z..

[53] Platis A (2020). Long-range modifications of the wind field by offshore wind parks—Results of the project WIPAFF. Meteorol. Z..

[54] Doms, G., Schättler, U. & Baldauf, M. *A Description of the Nonhydrostatic Regional COSMO Model*. *DWD COSMO V5.4*. http://www.cosmo-model.org (2011).

[55] Dee DP (2011). The ERA-Interim reanalysis: Configuration and performance of the data assimilation system. Q. J. R. Meteorol. Soc..

[56] Akhtar, N. & Chatterjee, F. Wind farm parametrization in COSMO5.0_clm15 (2020) doi:10.35089/WDCC/WindFarmPCOSMO5.0clm15.

[57] Akhtar, N. coastDat-3_COSMO-CLM_HR_WF. *World Data Center for Climate *(*WDCC*)* at DKRZ. *http://cera-www.dkrz.de/WDCC/ui/Compact.jsp?acronym=DKRZ_LTA_302_ds00001 (2020).

[58] Akhtar, N. coastDat-3_COSMO-CLM_HR. *World Data Center for Climate *(*WDCC*)* at DKRZ. *http://cera-www.dkrz.de/WDCC/ui/Compact.jsp?acronym=DKRZ_LTA_302_ds00002 (2020).

