# Author Correction: Accelerating deployment of offshore wind energy alter wind climate and reduce future power generation potentials

**DOI:** 10.1038/s41598-021-97055-3

**Published:** 2021-08-27

**Authors:** Naveed Akhtar, Beate Geyer, Burkhardt Rockel, Philipp S. Sommer, Corinna Schrum

**Affiliations:** Institute of Coastal Systems-Analysis and Modeling, Helmholtz-Zentrum Hereon, Geesthacht, Germany

Correction to: *Scientific Reports* 10.1038/s41598-021-91283-3, published online 03 June 2021

The original version of this Article contained errors.

In the Discussion and conclusions section,

“Depending on the size of the wind farm, generally, the annual mean wind speed deficit can reach 2–2.5 ms^−1^ which is equivalent to the power loss of 1–2 kWs^45^.”

now reads:

“Depending on the size of the wind farm, generally, the annual mean wind speed deficit can reach 2–2.5 ms^−1^ which is equivalent to the power loss of 1–2 MW^45^.”

In addition, Reference 45 was incorrectly given as:

Jonkman, J., Butterfield, S., Musial, W. & Scott, G. Definition of a 5-MW reference wind turbine for offshore system development. *Contract* 10.1002/ajmg.10175 (2009).

The correct reference is listed below:

Jonkman, J., Butterfield, S., Musial, W. & Scott, G. Definition of a 5-MW reference wind turbine for offshore system development. United States. 10.2172/947422 (2009).

The original Article has been corrected.

