# Supplementary material for: Accelerating deployment of offshore wind energy alter wind climate and reduce future power generation potentials
Source: Sci Rep. 2021 Jun 3;11:11826. doi: 10.1038/s41598-021-91283-3 (PMC8175401; doi:10.1038/s41598-021-91283-3)
Supplement: Supplementary file 1 — Supplementary Figures. [file 41598_2021_91283_MOESM1_ESM.pdf]

Supplementary information

Accelerating deployment of offshore wind energy alter wind climate and reduce future power generation potentials

Naveed Akhtar*, Beate Geyer*, Burkhardt Rockel*, Philipp S. Sommer*, Corinna Schrum*
* Institute of Coastal Systems -Analysis and Modeling, Helmholtz-Zentrum Hereon, Geesthacht, Germany

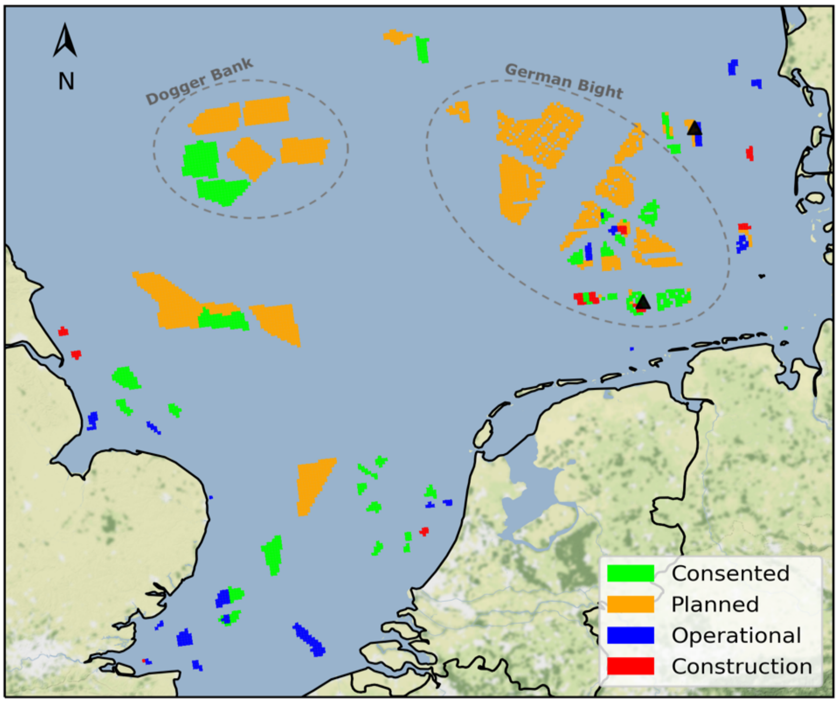

Figure SI 1. Model domain and distribution of OWFs in the North Sea (OWFs data from EWEA[1]). Colors indicate the planning status of the OWFs by 2015 in the North Sea and the land-sea-mask of the model domain. This figure was created with Matplotlib[2] and Cartopy[3].

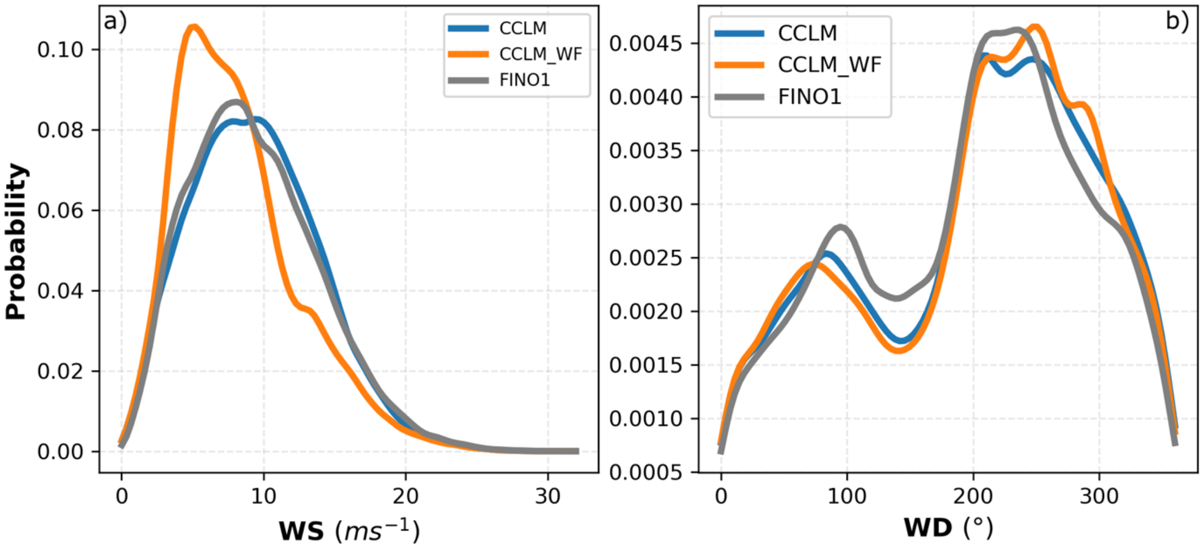

Figure SI 2. Probability density functions calculated using the mean hourly (a) wind speed and (b) wind direction for CCLM and CCLM_WF at the location of FINO1 (6.5875 °E and 54.01472 °N) in the period 2008–2017.

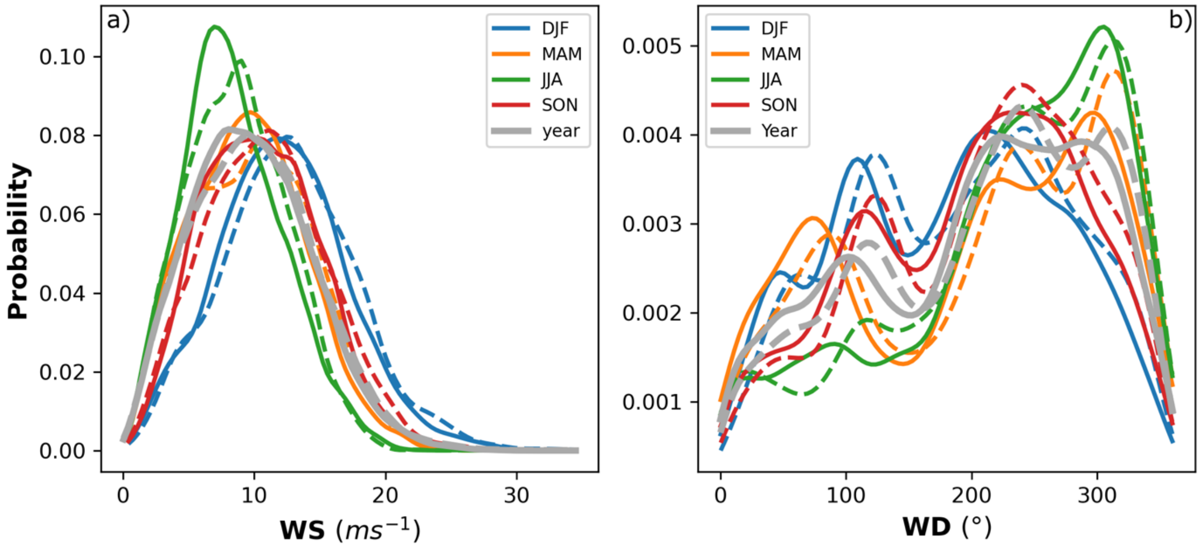

Figure SI 3. Annual and seasonal probability density functions calculated using the hourly (a) wind speed and (b) wind direction data at FINO3 (7.158333 °E, 55.195 °N) at a height of 90 m in the period 2008–2009. Dashed lines result from measurements, while solid lines are from the CCLM simulation. Gray lines indicate data for the entire period whereas colors indicate the different seasons as given in the legend.

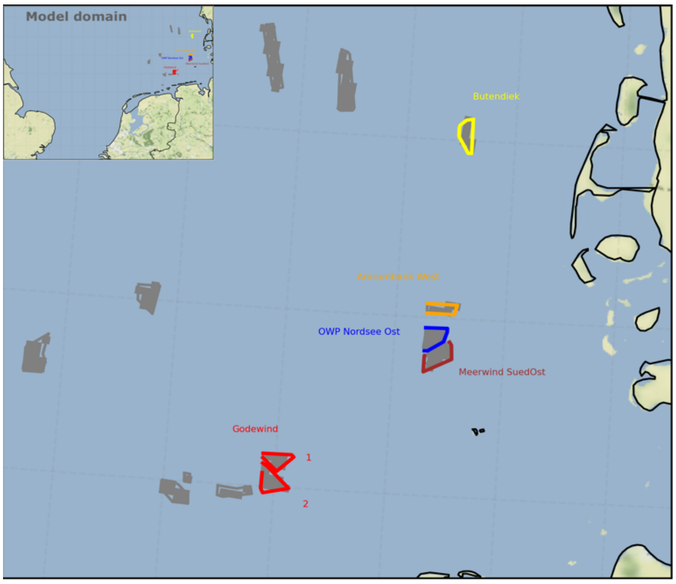

Figure SI 4. Distribution of operational offshore wind farms (gray polygons) by 2017[4] *in the German Bight area and model domain (top left). This figure was created with Matplotlib*[2] *and Cartopy*[3].

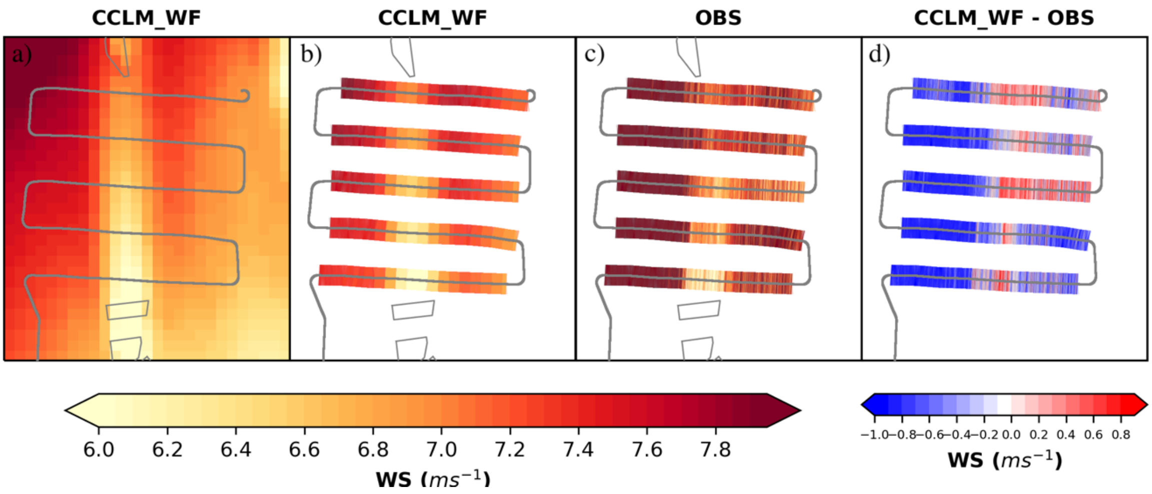

Figure SI 5a. Wind speed at a 90 m hub height (a and b) simulated in CCLM_WF, (c and d) observed and (d) difference between the CCLM_WF simulation and observation. The aircraft track (gray lines) shown here ranged from 0820 to 0924 UTC on 10 September 2016. The model simulations show the wind speed at 0900 UTC. This figure was created with Matplotlib[2] and Cartopy[3].

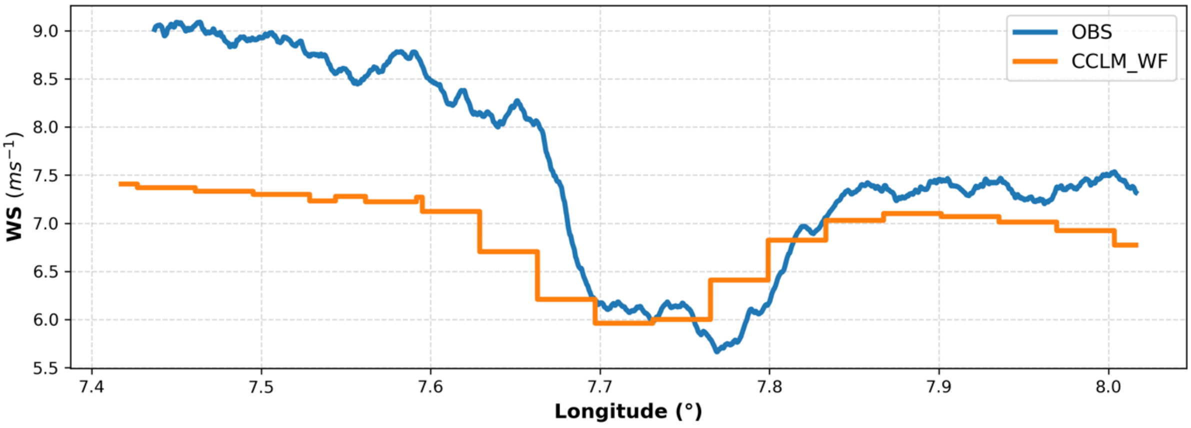

Figure SI 5b. Transect of the wind speed along first lap of the aircraft track shown in Fig. 3a.

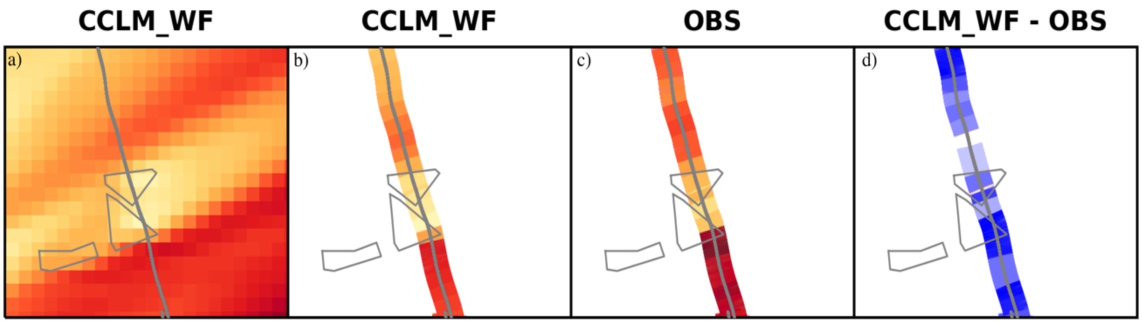

Figure SI 6a. Wind speed at a height of 250 m (a and b) simulated in CCLM_WF, (c) observed and (d) difference between the CCLM_WF simulation and observation. The aircraft track shown here ranged from 1445 to 1500 UTC on 14 October 2017. The model simulations show the wind speed at 1500 UTC. This figure was created with Matplotlib[2] and Cartopy[3].

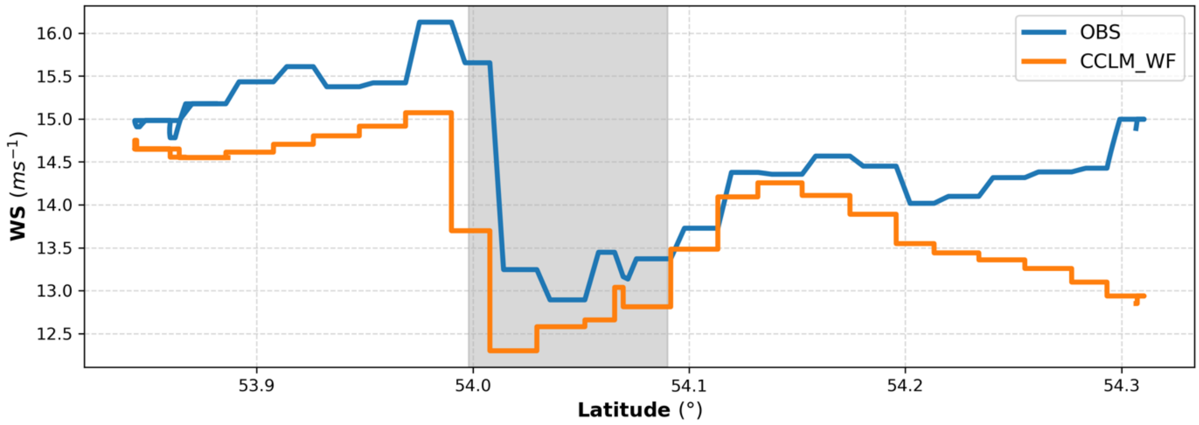

Figure SI 6b. Transect of the wind speed along the aircraft track shown in Fig. 4b. Gray areas denote the locations of wind farms.

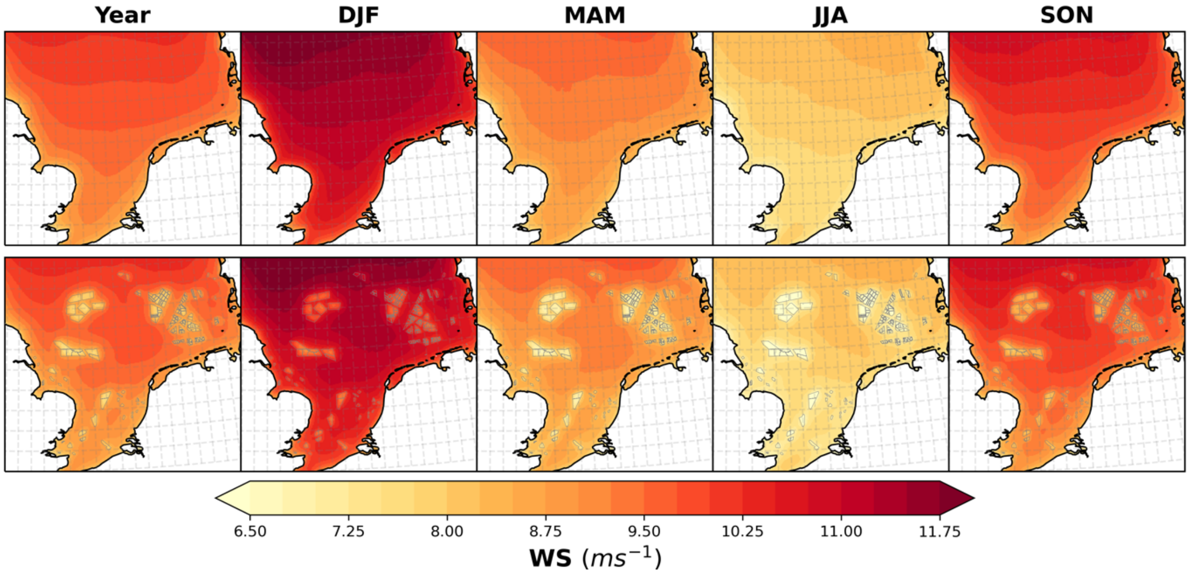

Figure SI 7. Annual and seasonal mean wind speeds simulated in CCLM (first row) and CCLM_WF (second row) at hub height (90 m) for wind directions of 0–360° in the period 2008–2017. This figure was created with Matplotlib[2] and Cartopy[3].

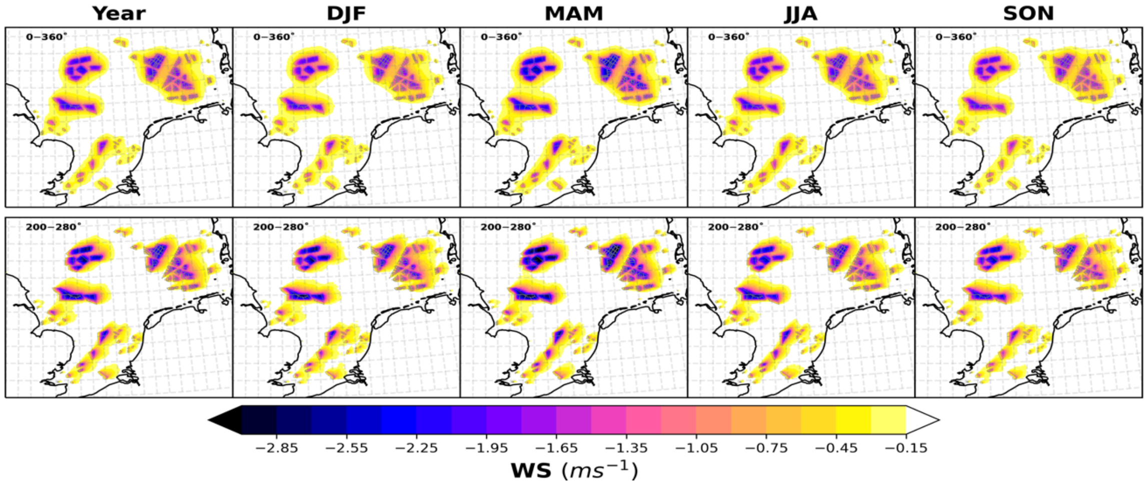

Figure SI 8. Annual and seasonal mean wind speed differences (CCLM_WF – CCLM) at hub height (90 m) for wind directions of 0–360° (first row) and 200–280° (second row) in the period 2008–2017. This figure was created with Matplotlib[2] and Cartopy[3].

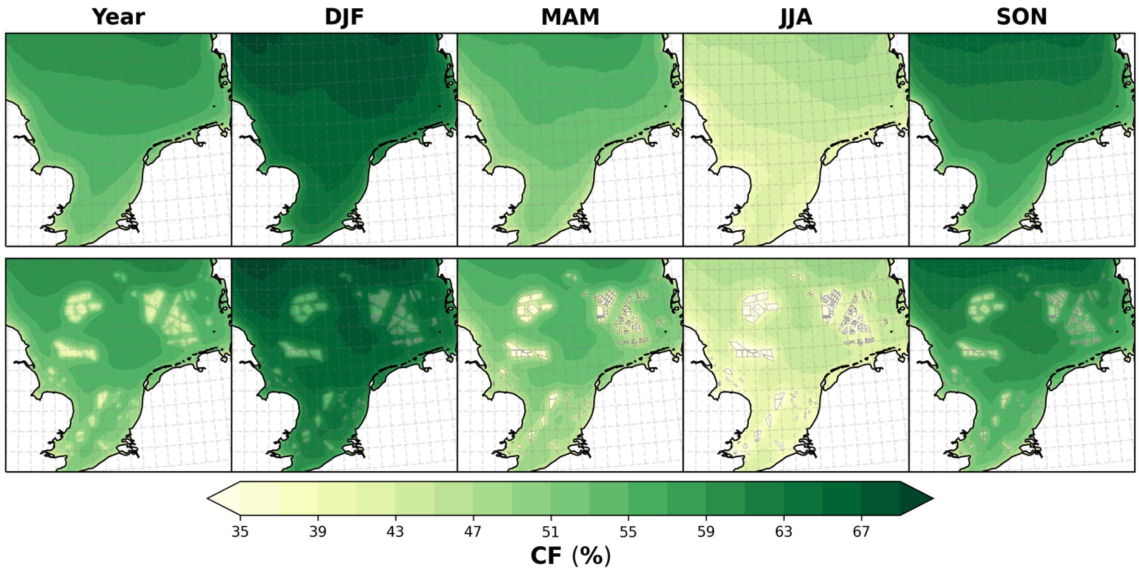

Figure SI 9. Annual and seasonal mean capacity factors calculated from CCLM (first row) and CCLM_WF (second row) at hub height (90 m) for wind directions of 0–360° in the period 2008–2017. This figure was created with Matplotlib[2] and Cartopy[3].

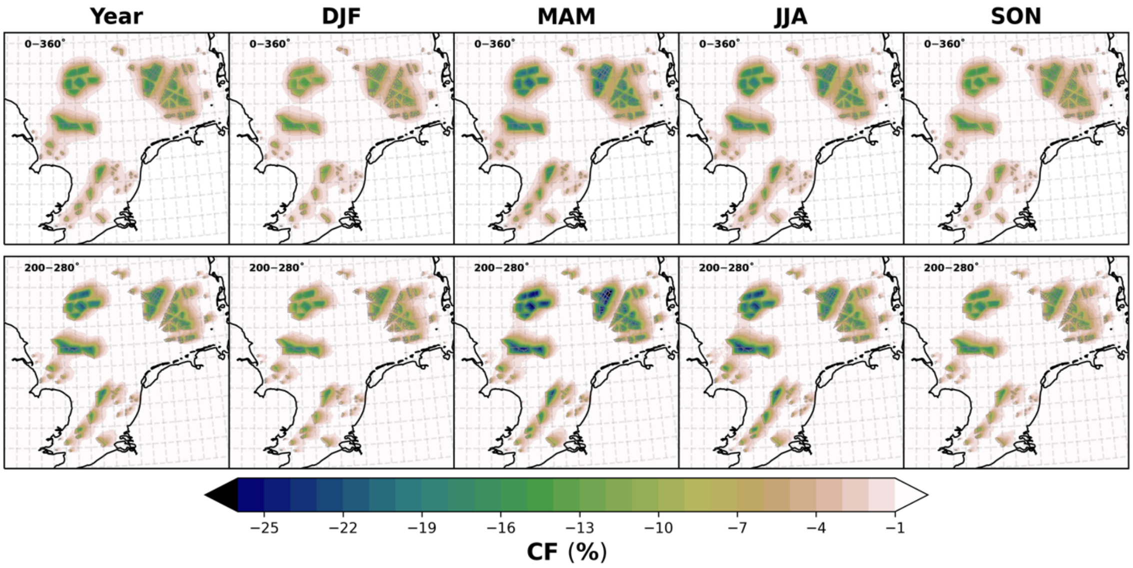

Figure SI 10. Annual and seasonal mean capacity factor differences (CCLM_WF – CCLM) at hub height (90 m) for wind directions of 0–360° (first row) and 200–280° (second row) in the period 2008–2017. This figure was created with Matplotlib[2] and Cartopy[3].

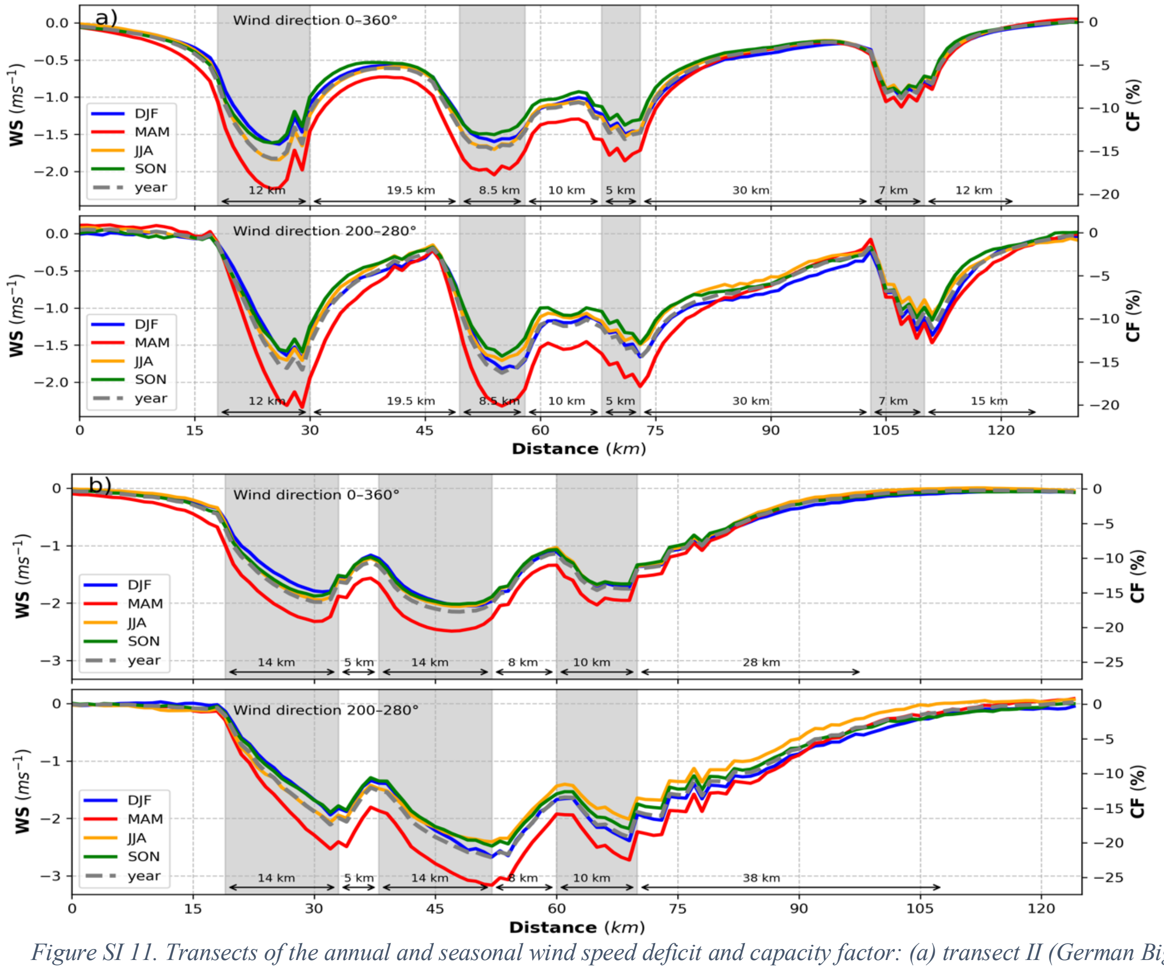

Figure SI 11. Transects of the annual and seasonal wind speed deficit and capacity factor: (a) transect II (German Bight, Fig. 2), 54.917 °N–54.24 °N and 4.85 °E–7.23 °E; (b) transect IV (Fig. 5) 54.33 °N–55.3 °N and 5.609 °E–7.30 °E. First row: all wind directions; second row: wind directions between 200 and 280° in the period 2008–2017. Gray areas denote the locations of wind farms. Arrows and attached numbers give the length of the wakes or distances between the edges of the wind farms respectively.

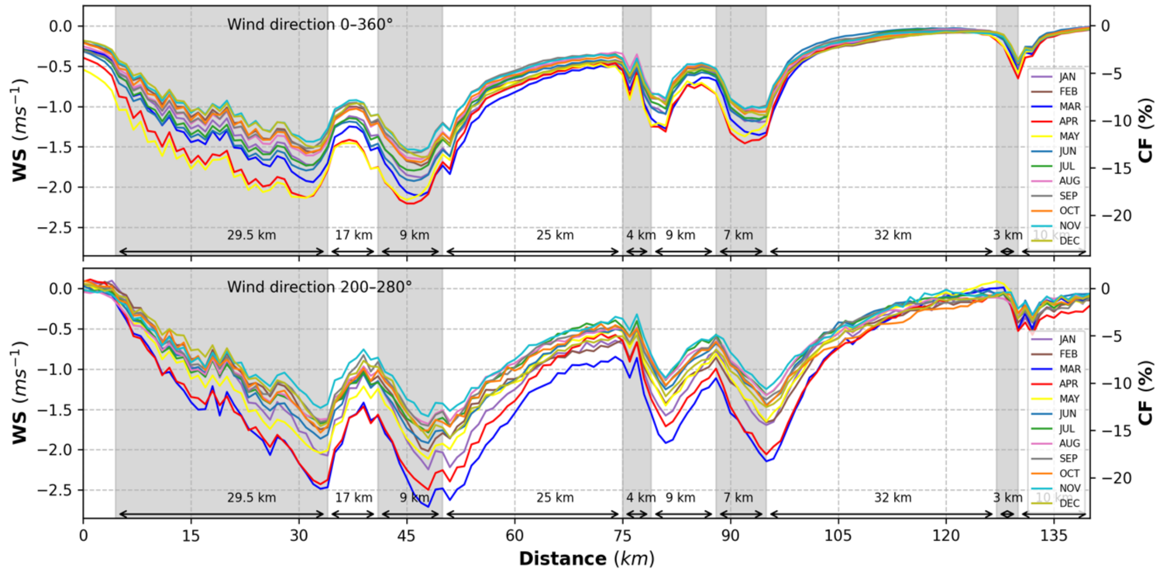

Figure SI 12. As of Fig. SI. 11 Transect I (Fig 5) the monthly mean wind speed deficit and capacity factor taken at 54.2 °N–55.6 °N and 5.45 °E–8.0 °E..

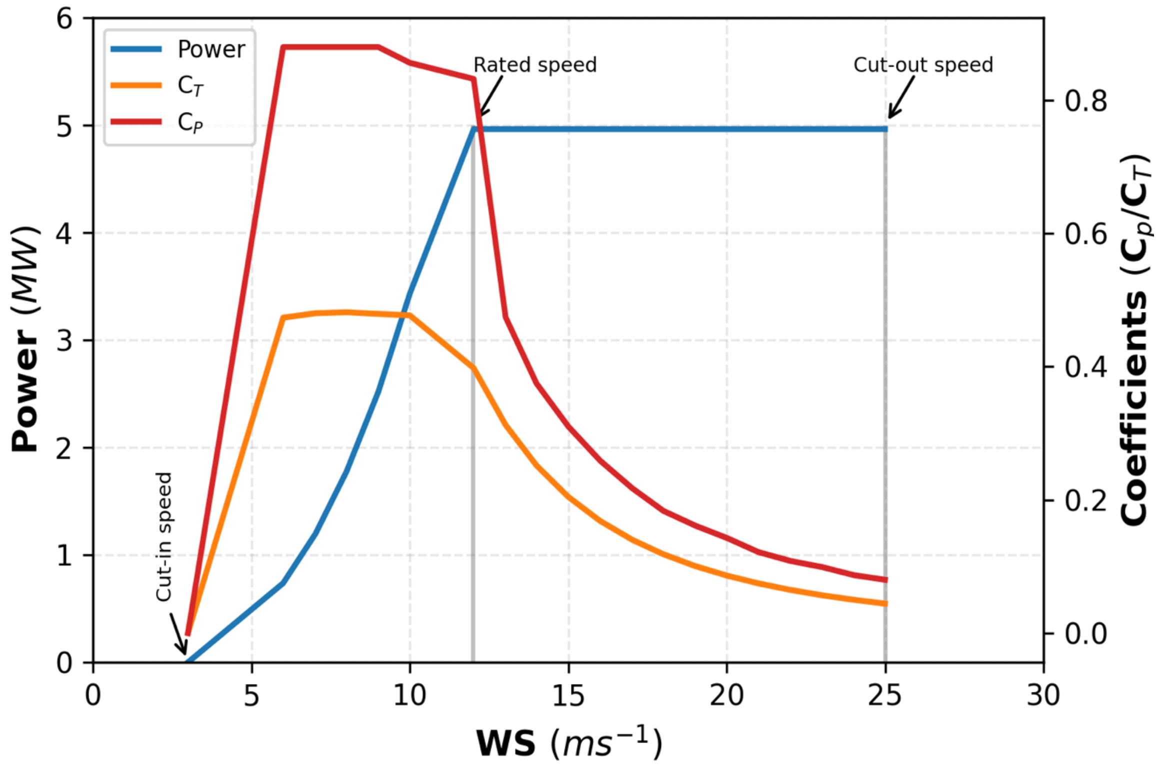

Figure SI 13. Curves of the power, thrust (C_T), and power coefficient (C_P) of the NREL 5 MW reference wind turbine.

1. EWEA. The European offshore wind industry key 2015 trends and statistics. ... 一 *Documents/Publications/Reports/Statistics/* ... 31 (2015) doi:10.1109/CCA.1997.627749.
2. Hunter, J. D. Matplotlib: A 2D graphics environment. *Computing in Science and Engineering* **9**, (2007).
3. Met office. Cartopy: a cartographic python library with a matplotlib interface. Exeter, Devon. https://scitools.org.uk/cartopy. (2015).
4. Platis, A. *et al.* First in situ evidence of wakes in the far field behind offshore wind farms. *Scientific Reports* **8**, (2018).
